# Treatment of children and adolescents with ulcerative colitis by adsorptive depletion of myeloid lineage leucocytes as monotherapy or in combination with low dose prednisolone after failure of first-line medications

**DOI:** 10.1186/1471-230X-13-130

**Published:** 2013-08-20

**Authors:** Tomotaka Tanaka, Shinichiro Sugiyama, Hirokazu Goishi, Tsuyoshi Kajihara, Morihisa Akagi, Toshio Miura

**Affiliations:** 1 Department of Gastroenterology, Akitsu Prefectural Hospital, 4388 Akitsu cho, Hiroshima 739-2402, Japan

**Keywords:** Paediatric inflammatory bowel disease, Ulcerative colitis, Adolescent, Prednisolone, Monotherapy, Myeloid lineage leucocyte, Adsorptive granulocyte and monocyte apheresis

## Abstract

**Background:**

Currently available drugs for the treatment of ulcerative colitis (UC) include salicylates, thiopurines, corticosteroids and new anti-tumour necrosis factor (TNF)-α biologics. Among these medications, corticosteroids in children and adolescents may adversely affect the patients’ growth and development. Further, UC patients have elevated and activated myeloid lineage leucocytes including the CD14 + CD16+ monocytes, which release TNF-α as a significant exacerbating factor. Accordingly, depletion of these cells by granulocyte/monocyte adsorption (GMA) should alleviate inflammation and promote UC remission. The objective of this study was to evaluate the efficacy of GMA in children and adolescents in whom conventional first-line medications had failed to induce remission.

**Methods:**

In a single centre setting, between 2007 and 2012, a total of 24 consecutive children and adolescents, age 11–19 years were given mesalazine or sulphasalazine as a first-line medication. Seventeen patients relapsed or did not respond to the first-line medications, and received GMA with the Adacolumn, 2 sessions in the first week, and then weekly, up to 11 sessions. Patients who achieved a decrease of ≥5 in the clinical activity index (CAI) were to continue with GMA, while non-responders were to receive 0.5 to 1.0 mg/kg/day prednisolone (PSL) plus additional GMA sessions similar to GMA responder cases. At entry and week 12, patients were clinically and endoscopically evaluated, allowing each patient to serve as her/his own control.

**Results:**

Seven patients achieved remission with the first-line medications and did not receive GMA. Five patients did not respond to the first 5 GMA sessions and received PSL plus GMA, while 12 patients responded to the first 5 GMA sessions and received additional sessions. At entry, the average CAI was 12.7 ± 2.5, range 8–17, and the average endoscopic index was 8.5 ± 1.5, range 7–11. The corresponding values at week 12 were 2.1 ± 0.2, range 1–4 (P < 0.001) and 2.4 ± 0.2, range 1–4 (P < 0.001). PSL was tapered to 0 mg within 3 months.

**Conclusions:**

With the strategy we applied in this study, all 24 consecutive patients achieved remission. In growing patients with active UC refractory to first-line medications, GMA was associated with clinical remission and mucosal healing, while in non-responders to GMA monotherapy, addition of a low dose PSL enhanced the efficacy of GMA and tapering of the PSL dose soon after remission was not associated with UC relapse. Therefore, the majority of young corticosteroid naive UC patients in whom first-line salicylates have failed may respond to GMA and be spared from additional drug therapy. Avoiding corticosteroids at an early stage of UC should ensure better long-term clinical course.

## Background

Ulcerative colitis (UC) is a debilitating chronic inflammatory bowel disease (IBD) that afflicts millions of individuals throughout the world and produces symptoms, which impair performance and quality of life [1]. Factors which initiate and perpetuate UC are not well understood yet. Accordingly, up to now medical therapy of UC has been empirical rather than based on a sound understanding of disease pathology. The empirical approach to therapy might in large part account for intolerance, treatment failure and adverse effects of pharmacologicals [2-4]. Historically, salicylates like sulphasalazine or 5-aminosalicylic acid have been used as the first-line medication for mild to moderate UC [1-3], while corticosteroids like prednisolone (PSL) are reserved for patients who do not respond to the first-line medication or develop acute severe UC [1,2,5]. Further, in children and adolescents with IBD, currently available medications like corticosteroids can adversely affect the patients’ growth and development [6], while for biologics, long term safety is unknown [7,8].

However, there is evolving evidence for peripheral myeloid lineage leucocytes like the CD14 + CD16+ monocytes as major sources of tumour necrosis factor (TNF)-α in patients with IBD [9,10]. Furthermore, UC patients may harbour elevated peripheral neutrophils as additional sources of TNF-α [11-13]. Indeed, neutrophil activation and prolonged survival is a feature of persistent intestinal inflammation and histological examination of the mucosal tissue reveals a spectrum of pathologic manifestations among which presence of an abundance of neutrophils accounts not only for the morphologic lesions of UC, but also for the prevailing patterns of mucosal inflammation [2,13]. Based on this background, the Adacolumn has been developed for selective depletion of granulocytes and monocytes (GM) by adsorptive apheresis (GMA). The column is filled with specially designed cellulose acetate beads of 2mm in diameter as the column leucocytapheresis carriers [14]. The carriers adsorb from the blood in the column most of the GM together with a significant fraction of platelets; lymphocytes are spared. In fact lymphocytes increase after GMA [14,15], including the CD4 + CD25 high + phenotype, which is known as a key regulator of immune function [16]. Here, we report on the efficacy and safety of GMA in paediatrics and adolescents with active UC in spite of receiving first-line medications.

## Methods

### Objective

Our major objective was to see if therapeutic granulocyte and monocyte adsorption (GMA) is effective as remission induction therapy in children and adolescents with active UC who were glucocorticosteroid naïve and had a short duration of UC (≤12 months). This was in line with our intention to avoid administering corticosteroids and other medications with safety concerns like cyclosporine and biologics to very young patients.

### GMA procedures

GMA with the Adacolumn is an established and officially approved extracorporeal treatment intervention for patients with active IBD in Japan [17-22]. Further, the Adacolumn conforms to the European standards (CE-marked) and therefore, in the countries of the European Union, its clinical application to treat IBD is based on the CE-mark [14,18-22]. In the present study, the Adacolumns were purchased from JIMRO (Takasaki, Japan) and used as previously described [14-19]. Briefly, the Adacolumn is a single use medical device filled with specially designed cellulose acetate beads of 2 mm in diameter, which serve as the column adsorptive leucocytapheresis carriers for FcγR and complement receptor bearing leucocytes. Therefore, the carriers selectively adsorb from the blood in the column most of the granulocytes, monocytes/macrophages and a significant fraction of platelets; lymphocytes are spared and subsequently increase [14,15]. Blood access was via a venipuncture in the antecubital vein in one arm and from the column outflow, blood return to the patient was via a venipuncture in the contralateral arm. The duration of one GMA session was 60 minutes, at 30 ml/minute. An optimum dose of sodium heparin (2000 units/session) was administered during GMA as an anticoagulant. In the present study, each patient was to receive 2 sessions in the first week and then weekly, up to 11 sessions in an outpatient setting.

### Treatment design and efficacy assessment

A total of 24 children and adolescents with a definitive diagnosis of UC were included in this investigation. The patients’ major demographic variables are presented in Table 1. These were all consecutive steroid naive patients received at our IBD unit between 2007 and 2012. Patients’ UC clinical activity index (CAI) was determined at baseline, prior to each GMA session and then within one week post last session according to Rachmilewitz [23]. CAI ≤4 meant clinical remission. Similarly, patients’ UC disease activity index (DAI) was determined based on colonoscopy done at entry, after 5 GMA sessions and then within one week following the final session or week 12 according to Sutherland, et al. [24]. Endoscopic remission meant DAI <3 and absence of blood in stool.

**Table 1:** Baseline demographic varialbles of 24 children and adolecents who responded to salicyte, GMA or GMA + prednisolone (PSL)

**Sub-group**	**Age**	**Gender**	**Weight (Kg)**	**UC location**	**UC duration (month)**	**CAI**	**DAI**	**Baseline medication**
Responded to salicylate alone (n = 7)	17	F	37.5	left side	3	10	7	Mesalazine, 80 mg/kg/day
	16	F	47.2	total side	3	11	8	Mesalazine, 64 mg/kg/day
	13	M	33.8	total colitis	6	10	7	Mesalazine, 89 mg/kg/day
	18	M	48.0	rectal	4	8	6	Mesalazine, 63 mg/kg/day
	17	M	55.5	rectal	6	9	7	Mesalazine, 72 mg/kg/day
	17	F	48.8	left side colitis	3	10	8	Mesalazine, 82 mg/kg/day
	11	F	33.0	left side colitis	1	10	7	Mesalazine, 90 mg/kg/day
Responded to GMA alone (n = 12)	14	M	41.7	total colitis	10	17	10	Sulphasalazine, 72 mg/kg/day
	16	F	41.8	left side colitis	6	13	9	Mesalazine, 72 mg/kg/day
	17	F	42.5	left side colitis	12	14	8	Mesalazine, 71 mg/kg/day
	11	F	41.5	left side colitis	10	13	7	Mesalazine, 72 mg/kg/day
	17	M	55	left side colitis	14	14	7	Sulphasalazine, 57 mg/kg/day
	15	M	40.6	left side colitis	6	14	10	Sulphasalazine, 74 mg/kg/day
	16	M	45.2	total colitis	6	13	9	Sulphasalazine, 66 mg/kg/day
	12	F	41.2	left side colitis	4	14	9	Mesalazine, 73 mg/kg/day
	15	F	34.2	left side colitis	6	13	10	Mesalazine, 55 mg/kg/day
	14	F	41.2	left side colitis	10	13	8	Mesalazine, 73 mg/kg/day
	19	F	48.0	left side colitis	6	11	8	Mesalazine, 83 mg/kg/day
	17	F	44.8	total colitis	1	10	7	Mesalazine, 89 mg/kg/day
Responded to GMA + PSL (n = 5)	12	M	34	total colitis	12	16	11	Sulphasalazine, 59 mg/kg/day
	13	F	31.5	left side colitis	6	14	10	Sulphasalazine, 63 mg/kg/day
	17	F	50	total colitis	6	16	11	Mesalazine, 60 mg/kg/day
	15	F	41	total colitis	6	16	10	Sulphasalazine,73 mg/kg/day
	17	F	42.6	total colitis	8	15	10	Mesalazine, 70 mg/kg/day

*CAI*, clinical activity index; *DAI* endoscopic disease activity index; GMA, granulocyte/monocyte adsorption; *M*, male; *F*, female; *UC* ulcerative colotis.

**Figure 1:**
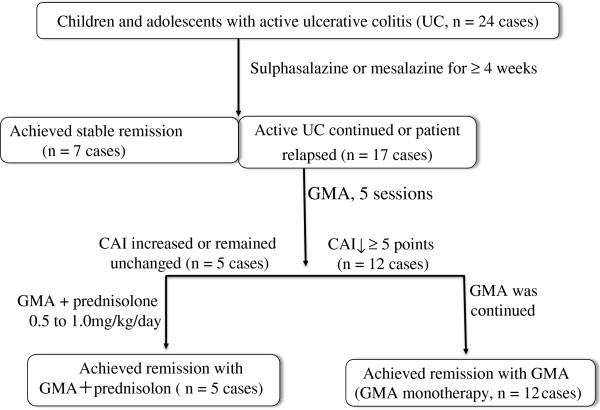
Study design and the treatment outcomes in 24 children and adolescents with active ulcerative colitis.

As seen in Table 1, the duration of UC was short in these cases. Following the diagnosis of UC, all patients were given a salicylate (sulphasalazine or mesalazine, 2000-4000 mg/day) as the first-line medication. The treatment design including the introduction of GMA for these patients is shown in Figure 1. Any patient who achieved remission with the first-line salicylate over a period of at least 4 weeks was to continue with that medication, while patients who were with active disease or had relapsed while on salicylate were selected for GMA. Further, it was decided that patients who achieved a decrease of ≥5 points in the CAI following 5 GMA sessions should continue with GMA, while non-responders were to be given 0.5 - 1.0 mg/kg bodyweight/day PSL orally plus additional GMA, up to 11 sessions without interrupting the GMA therapy. This was to allow us assessing the efficacy of GMA in growing patients as monotherapy or as a combination intervention. However, PSL was to be tapered at 5 mg/week when a patient showed an improvement in the CAI score by at least 5 points.

### Ethical considerations

All treatment interventions applied in this study including GMA are officially approved, and are routine therapeutic options for patients with active IBD in Japan. Accordingly, prior to the initiation of the treatments, this work was approved by ethic committees at the Hiroshima Chugoku Rosai and Akitsu hospitals. Additionally, informed consent was obtained from all patients after explaining the study aim and the nature of the procedures involved. In all under age cases, consent from one of the patients’ parents was obtained. Further, adherence was made to the Principle of Good Clinical Practice and the Helsinki Declaration at all times.

### Statistics

Where appropriate, data are presented as the average (mean ± SD) values together with ranges. The overall CAI and DAI scores at baseline and post GMA are compared by using the paired t-test. Comparison of data sets in sub-groups was done by using the Steel-Dwass test. All statistical analyses were done by using the software JMP, version 10.1 (SAS Japan). P < 0.05 was considered statistically significant.

## Results

### Patients’ demographic variables

Among the 24 children and adolescents included in this report (Table 1), 8 were female. Average age was 15.3 ± 2.3 years, range 11–19 years, average weight was 42.5 ± 6.5 kg, range 31.5-55.5 kg, and average duration of UC from the time of diagnosis to the first GMA session was 6.46 ± 3.41 months, range 1 month (shortest) to 12 months. Regarding the severity of UC, average CAI was 12.7 ± 2.5, range 8–17, and average DAI was 8.5 ± 1.5, range 7–11. Therefore, UC was moderately active in the majority of the 24 patients.

### Efficacy based on CAI and DAI

**Figure 2:**
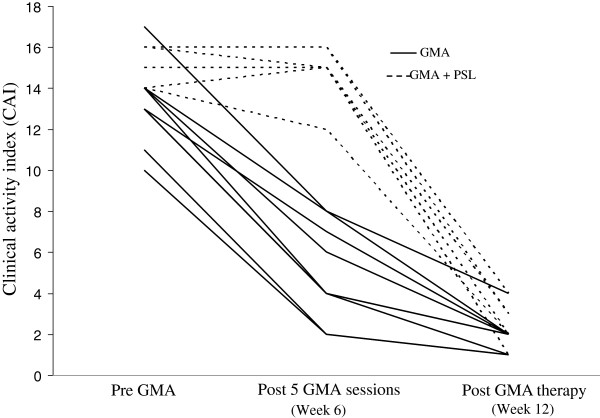
**Clinical activity indices in 17 children and adolescents at entry, after 5 adsorptive granulocyte and monocyte apheresis (GMA) sessions and then one week post final session (or week 12).** Solid lines represent patients who achieved remission with GMA, while dotted lines indicate patients received 0.5 to 1.0 mg/kg bodyweight/day prednisolone (PSL) in combination with GMA. All 17 patients achieved clinical remission (CAI ≤4).

**Figure 3:**
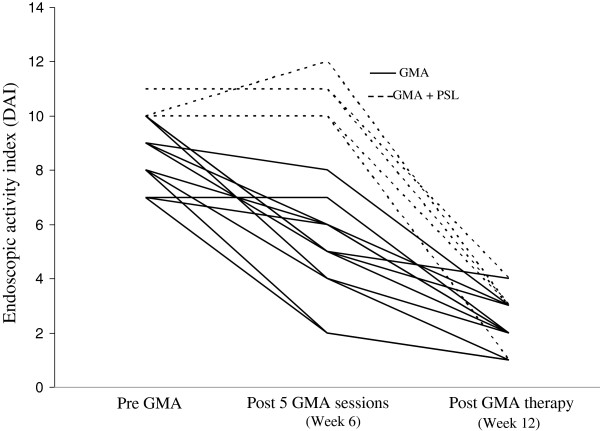
**Endoscopic disease activity indices (DAI) in 17 children and adolescents at entry, after 5 adsorptive granulocyte and monocyte apheresis (GMA) sessions and then one week post final session (or week 12).** Solid lines represent patients who responded to GMA without any additional medication, while dotted lines indicate patients received 0.5 to 1.0 mg/kg bodyweight/day prednisolone (PSL) in addition to GMA. All 17 patients had improvement of DAI including 9 with complete remission.

Figure 1 shows that 7 of 24 patients achieved clinical remission (CAI ≤4) while on sulphasalazine or mesalazine. These 7 patients were not included for GMA therapy. The CAI measurements at entry and post GMA therapy for the 17 patients who received GMA are presented in Figure 2, while the corresponding DAI measurements are presented in Figure 3. The average CAI scores prior to GMA and post final session were 12.7 ± 2.5, range 8–17 and 2.1 ± 0.2, range 1–4 (P < 0.001), respectively. Only in one case, CAI was 4 at post GMA therapy, but smaller than 4 in 16 patients. Likewise, DAI values prior to the first GMA session and at the end of GMA course were 8.5 ± 1.5, range 7–11, and 2.4 ± 0.2, range 1–4 (P < 0.001), respectively. DAI was <3 in 9 cases (endoscopic remission), equal to 3 in 6 cases and 4 in 2 cases. Therefore, all 17 GMA-treated patients achieved clinical remission including 9 with complete remission (mucosal healing).

As shown in Figure 1, 5 patients did not respond to the first 5 GMA sessions and were given GMA plus PSL, but the PSL dose was tapered to 0mg within 3 months. Therefore, among 17 patients in the GMA group, 12 achieved remission with GMA as monotherapy and 5 patients achieved remission with GMA in combination with PSL.

**Figure 4:**
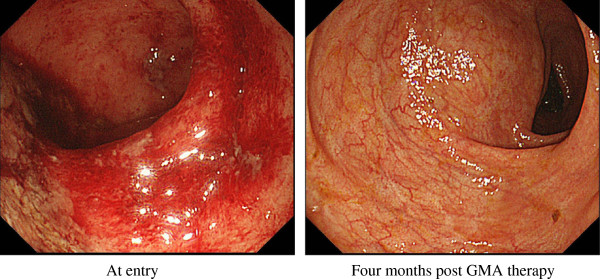
**Typical endoscopic finding at entry and post GMA course in a patient who achieved complete remission by GMA as monotherapy.** This case, in spite of showing strong inflammation in the mucosa and contact bleeding, responded well to GMA with fully restored mucosal vascular patterns.

Previously, we reported that patients with deep ulcers together with extensive loss of the mucosal tissue at the lesion sites were not likely to benefit from GMA [25]. With this in mind, colonoscopy was done at baseline in all patients who were to receive GMA therapy. As reported above, these 24 patients had a short duration of UC and none showed badly damaged mucosa, and this is reflected in high clinical efficacy and endoscopic mucosal healing rates in these patients. One typical colonoscopy image is presented in Figure 4. This case, in spite of showing strong inflammation in the mucosa and contact bleeding, responded well to GMA.

### Sub-group analyses

As stated above, 7 patients achieved remission with salicylate as the sole first-line medication (n7), 12 achieved remission with GMA as monotherapy (n12), and 5 patients achieved remission with GMA + PSL (n5). We were interested to see if baseline demographic variables in these subgroups could predict the observed clinical response. Prior to initiation of GMA, CAI measurements in these sub-groups were 9.7 ± 0.9 (n7), 13.3 ± 1.2 (n12), and 15.4 ± 0.9 (n5). The statistical significance levels were as follows: n7 vs n12 (P < 0.01); n7 vs n5 (P < 0.01); n12 vs n5 (P = 0.04). Similarly, the average DAI scores in n7, n12 and n5 subgroups were 7.1 ± 0.7, 8.5 ± 1.2 and 10.4 ± 0.6, respectively. The significance levels were: n7 vs n12 (P = 0.05, not significant); n7 vs n5 (P = 0.01); n12 vs n5 (P = 0.02). Based on the statistical analyses we carried out, patients who responded to the first-line mesalazine or sulphasalazine alone had a significantly lower average CAI as compared with patients who received GMA. Likewise, patients who achieved clinical remission with GMA as monotherapy had significantly lower average CAI core as compared with patients who achieved remission after receiving GMA + PSL.

### Maintenance rate of remission

The average follow-up time after the last GMA session was 29.9 months, range 5 to 80 months. At the time of this writing (January 2013), 15 of 24 patients were still in remission (62.5%) with the salicylates as the only ongoing medications. These were 7 of 7 cases in n7 (average 15 months), 6 of 12 cases in n12 (average 8.8 months) and 2 of 5 cases in n5 (average 10.3 months). This echoes the outcome in connection with baseline CAI and the clinical response to first-line medications, GMA monotherapy and GMA + PSL.

### Treatment safety, feasibility and patients’ compliance

In general, GMA with the Adacolumn in our hospital is very much favoured by our patients for its safety feature. Given that the patients in this report were all young, we had to show extra vigilance to monitor side effects. As the reality of clinical practice would suggest, venous access in children is not as straightforward as in an adult patient. Accordingly, blood access was a serious challenge and needle pain was experienced by 8 of 17 patients (47.1%). Other transient side effects were mild headache in 8 patients, nausea and light headedness in 6 patients (35.3%), vomiting in 4 patients (23.5%). However, these adverse events were not serious and all 17 cases completed their 11 GMA sessions according to the treatment schedule, compliance was 100%. Further, although GMA carriers adsorb a large fraction of ganulocytes and monocytes from the blood which passes through the Adacolumn, but the levels of these cells in the peripheral circulation were found not to fall significantly [14] due to an influx of naive leucocytes (CD10 negative neutrophils) from the marginal pools including the bone marrow into the circulation [14]. The naive leucocytes are thought to be less inflammatory [26]. Therefore, neither in this study nor in previous GMA studies [10,14-22], patients showed opportunistic infection.

## Discussion

Regarding GMA as a therapeutic intervention in patients with UC, it is of paramount importance to know that clinical efficacy outcome reports vary from an 85% [25,27,28] to a statistically insignificant level [29]. Regardless of the outcome in a controlled study [29], our own experience while treating over 200 patients during the past 12 years indicates that first episode cases as well as steroid naïve patients, notably those with a short duration of UC respond well to GMA and are spared from drug therapy [25,27,28,30]. It should be appropriate to mention here that Yamamoto and colleagues [17] reported that patients who responded to GMA had a better long-term clinical outcome by avoiding corticosteroids during their first active UC phase. In contrast, patients with deep mucosal lesions and extensive loss of the mucosal tissue at the lesion sites together with those who have a long duration of active UC and exposure to multiple conventional drugs including corticosteroids are not likely to benefit from GMA [25,29-31]. The demography of the young patients in this report was consistent with the GMA responder features. Accordingly, we thought that these patients should respond well to GMA and be spared from additional pharmacologic intervention. The therapeutic outcomes in these 24 cases might be summarised as follows.

Following the diagnosis of UC, every one of these children/adolescents was given a salicylate as the first-line medication. Among the 24 patients, 17 developed active disease while on a salicylate or did not respond to the first-line medications. Most of the 17 patients did respond to GMA and avoided additional pharmacologic therapy. Only in a minority of the 24 patients corticosteroid was used, which was discontinued when a patient showed improvement. Therefore, 3 months after the initiation of GMA therapy, all 24 young patients were in clinical remission, and none was on a corticosteroid. As reported above, the efficacy of GMA is intimately related to patients’ endoscopic findings at entry [25,29,30]. With this in mind, colonoscopy was done at baseline in all patients who were to receive GMA. However, as mentioned above, these 24 patients had a short duration of UC and none showed badly damaged mucosa, and this is reflected in a high clinical efficacy and endoscopic mucosal healing rates in these patients. Generally, GMA has a good safety profile, and therefore, apart from needle pain in a significant fraction of these youngsters, we had no serious safety concern to report here. In spite of the needle pain, all 17 patients completed their GMA course according to the protocol.

A recent expert report [32] recommends that first episode cases or patients with a single inflammatory exacerbation in a 12-month period should receive 5-aminosalicylate instead of corticosteroids due to safety concerns. Adverse effects of drug based medications in very young patients can be a serious addition to the morbidity associated with IBD. Fortunately, it appears that drug naïve patients respond well to GMA. Our own experience suggests that the best time to apply GMA is immediately after a clinical relapse, which means a short duration of active disease [31]. As GMA is a non-drug intervention, it is unlikely to cause refractoriness.

At this point, we like to make specific comments on the merit and limitations of this report. Here we describe the outcomes in 24 young patients who were treated in a consecutive setting over a period of 5 years. Apart from being an open study, 5 patients who did not improve after the first 5 GMA sessions received PSL, while GMA was continued. Therefore, we can not claim that these 5 cases might not have achieved remission with PSL alone. However, as stated above, our objective was to avoid corticosteroids in these young patients. The 19 patients who did not receive corticosteroids should have a better long-term clinical course as reported for adult patients [17]. With this in mind, PSL was tapered to 0mg when a patient improved, and this did not provoke a relapse. Likewise, our subgroup analyses showed that patients with a lower entry CAI responded well to the first-line medications or GMA monotherapy, while those with a significantly higher CAI score needed PSL. In the same order, patients had better maintenance rate of remission (see Results section). We are aware that these subgroups had a small number of cases and further studies in larger cohorts of patients are expected. Additionally, we have referred to patients who responded to GMA without adding any other medication as GMA monotherapy. One could logically argue that those patients were on concomitant salicylates. However, except in one case, salicylates had been continued for at least 4 weeks prior to GMA in 11 of the 12 GMA monotherapy cases and all 17 GMA-treated patients had relapsed or had active disease while on a salicylate. We believe that the superiority of GMA over corticosteroids warrants to be shown in a future randomised controlled trial in large cohorts of paediatric UC patients in whom first-line medications have failed; patients in one arm should receive GMA, while patients in another arm receive corticosteroid as remission induction therapy.

## Conclusions

In this study, children and adolescents with UC who were corticosteroid naïve and had a relatively short duration of UC were given first-line medications and those who did not achieve remission were given GMA as a non-pharmacologic treatment intervention. Of 24 patients only 5 patients received corticosteroid, but only over a short period of time. GMA was associated with clinical remission and mucosal healing. Therefore, the majority of young UC patients in whom first-line salicylates have failed may respond well to GMA and be spared from corticosteroids and other pharmacologics with safety concern. Further, in our hospital, GMA is very much favoured by patients for its safety profile and being a non-drug intervention. This is of significant clinical interest in children in whom long-term drug based intervention can adversely affect the patient’s growth and development. A future randomised controlled trial in large cohorts of paediatric UC patients should strengthen the findings of this study.

## Abbreviations

CAI: Clinical activity index; DAI: Disease activity index; FcγR: Fragment crystallizable gamma receptor; GM: Adsorptive granulocytes and monocytes; GMA: Granulocyte and monocyte adsorptive apheresis; IBD: Inflammatory bowel disease; PSL: Prednisolone; TNF: Tumour necrosis factor; UC: Ulcerative colitis.

## Competing interest

The authors declare having no conflict of interest in connection with this publication.

## Authors’ contributions

TT: Conception, study design, and drafting of the final manuscript version. TT, SS, HG, TK, MA, TM: Patient management, acquisition, interpretation of the data, statistics, and review of the final manuscript draft. All authors read and approved the final manuscript.

## Pre-publication history

The pre-publication history for this paper can be accessed here:

http://www.biomedcentral.com/1471-230X/13/130/prepub
